# Identifying Cognitive States Using Regularity Partitions

**DOI:** 10.1371/journal.pone.0137012

**Published:** 2015-08-28

**Authors:** Ioannis Pappas, Panos Pardalos

**Affiliations:** 1 Department of Industrial and Systems Engineering, College of Engineering, University of Florida, Gainesville, Florida, United States of America; 2 Department of Biomedical Engineering, College of Engineering, University of Florida, Gainesville, Florida, United States of America; University Of Cambridge, UNITED KINGDOM

## Abstract

Functional Magnetic Resonance (fMRI) data can be used to depict functional connectivity of the brain. Standard techniques have been developed to construct brain networks from this data; typically nodes are considered as voxels or sets of voxels with weighted edges between them representing measures of correlation. Identifying cognitive states based on fMRI data is connected with recording voxel activity over a certain time interval. Using this information, network and machine learning techniques can be applied to discriminate the cognitive states of the subjects by exploring different features of data. In this work we wish to describe and understand the organization of brain connectivity networks under cognitive tasks. In particular, we use a regularity partitioning algorithm that finds clusters of vertices such that they all behave with each other almost like random bipartite graphs. Based on the random approximation of the graph, we calculate a lower bound on the number of triangles as well as the expectation of the distribution of the edges in each subject and state. We investigate the results by comparing them to the state of the art algorithms for exploring connectivity and we argue that during epochs that the subject is exposed to stimulus, the inspected part of the brain is organized in an efficient way that enables enhanced functionality.

## Introduction

Studying the human brain has gained significant interest the past years due to the advances of neuroimaging techniques. A typical fMRI study is delineated as follows: Fluctuations in the oxygen levels of the blood (BOLD signals) are captured usually in low frequencies (0.01–0.05 Hz) with significant spatial resolution [1]. In particular, when recording, fMRI images are being drawn usually every 0.5 or 1 second. Each image consists of a collection of voxels that are rudimentary volume elements of the cortex (usually few cubic millimeters). Each image can contain thousands of voxels whereas each voxel may reflect the activity of a large group of neurons.

The temporal resolution of fMRI is considered over several seconds where each second may consist of a large amount of images. Consequently, each voxel can be described by a specific BOLD response over time.

Machine learning techniques have been used to describe cognitive states using the aforementioned sequence of images [2, 3]. There has been significant work using trained classifiers. A significant drawback of these techniques is their inability to describe the organization, topology, and dynamics of the brain during different states.

A popular trend in exploring brain’s functional connectivity is to model and analyze the brain in terms of a network. In general this approach consists of modeling the brain as graph and, in conjunction with fMRI data, nodes and edges are represented in terms of voxels and their pairwise correlations. There has been a significant amount of research towards exploring topological properties of different brain networks [4], how networks and clustering are associated with different states [5], and how effective algorithms can be implemented in order to assess these [6].

In this study, we turn our attention to data collected during a specific cognitive experiment and we focus on three points that stand on the intersection of the previous remarks:

1. We wish to identify differences in functional connectivity between during different cognitive states. We plan to use our new algorithm in order to identify differences that in statistically similar networks.
2. We wish to explore the modularity of these networks using established methods for community detection and their internal organization. We intend to use our new algorithm in a way that will enable to identify any hidden organization in the network.
3. We wish to use our algorithm to reduce the network data. Reducing the data will enable us not only to gain efficiency in terms of computational load but also to compare application of the state-of-art clustering algorithms to the reduced data as opposed to a direct application on the raw data.

Identifying differences in functional connectivity networks during various states and subjects has already been established as a part of a tool called the Network-based statistic [6]. In addition, organization and modularity of brain networks, as clustering techniques can reveal, have already been introduced [7] in parallel to some novel algorithms for identifying hierarchical organization [8].

Our most important tool in this analysis will be a novel clustering technique that we will call regularity partitioning algorithm. Its basis lies on the regularity lemma used by Szemerédi [9, 10, 11] to prove his theorem about the existence of arithmetic progressions in dense sets. Its powerful nature stands on its universality; every graph of sufficient size can be partitioned into clusters that behave as pseudo-random pairs. Randomness [12, 13] can be associated with the existence of as many edges as possible in a graph. Using appropriate definitions, we will be able to identify pairs of clusters such that their number of edges between them is highly increased. In turn, this will enable us to quantify the existence of patterns in the graph and draw conclusions about how the edges are distributed. Although, its theoretical strength, it has not been considered for practical applications mainly due to the fact that it requires graphs of astronomically large size. Sárközy et al. removed this impediment in [14], where they introduced a practical partitioning algorithm that performs for smaller graphs as well. As we shall see in the results, its importance lies on the fact that all the clusters of vertices identified by the algorithm are pseudo-random with each other. In this work, we seek to apply this algorithm to the brain networks constructed at three different cognitive stages of two subjects. We are going to depict the results and provide basis for the functional connectivity of the brain by quantifying the distribution of edges, and discuss the implications that this methodology has to the discrimination of cognitive states. In addition, we are going to reduce the data based on this clustering technique and we are going to compare our results with existing algorithms.

## Materials and Methods

### Preliminary analysis

For this work, we considered data from two subjects from the study described in [2]. In this study patients were being showed a sentence followed by an arrangement of symbols two symbols (symbol1 and symbol2). The symbols that were used were“+”, “$”, and “*”. The sentences were of the form “it is true that symbol1 is above/below symbol2” (case 2) or of the form “it is not true that symbol1 is above/below symbol2” (case 3) followed by an arrangement of the symbols relative to the sentence (for instance $\frac{+}{*}$). Eventually, the subjects had to answer the question presented in the first stimulus. The first stimulus was presented to the subjects for 4 seconds, followed by a blank screen for another 4 seconds. The second stimulus was presented for a maximum time of 4 seconds; this part ended when the subject responded to whether the sentence was appropriately describing the arrangements. A relaxation period of 15 seconds was the last part of the trial before the next trial began. Thus, the total duration of the trial was approximately 27 seconds. fMRI data were collected every 0.5 seconds. 6 subjects were used to conduct this study where each subject went under 54 trials as described above (with 8 fixations periods interpolated–case 1). The Carnegie Mellon Institutional Review Board (IRB) approved this research and collection of data for this study. Patient records/information was anonymized and de-identified prior to analysis.

Data collected from this study were processed and stored as.mat files that are ready to being processed by MATLAB (the MathWorks, Inc., Natick, MA, US.). No additional smoothing took place. For each subject, the data consisted of time series corresponding to the fMRI activity of each voxel. Therefore for each subject, the dimension of the data was number_of_trials × number_of_voxels × duration_each_trial.

In order to assess the functional connectivity of the brain we used correlation between voxels. In specific, we constructed the affinity matrix of the voxels as follows: For each pair of voxels *X* and *Y* we calculated the correlation between them as

$$r_{XY}=\frac{\sum_{i=1}^{T}{(X}_{i}-\bar{X})(Y_{i}-\bar{Y})}{\sqrt{\sum_{i=1}^{T}{{(X}_{i}-\bar{X})}^{2}}\sqrt{\sum_{i=1}^{T}{{(Y}_{i}-\bar{Y})}^{2}}}$$

where *T* is the total number of observations and $\bar{X}$ and $\bar{Y}$ are the averages of the signals. The adjacency matrix M was constructed as follows:

$$M(i,j)=1\;if\;r_{ij}(f)>threshold\;and\;0\;otherwise.$$

where the threshold was chosen adaptively in order to keep the 10% of the most significant connections. Building adjacency matrices based on the biomedical data using different interdependence measures is a well-examined technique [15, 16, 17]. The threshold for fathoming weights was set under the perspective to try to keep the most meaningful edges based on their respective histogram [18]. Correlation though suffers from flaws just as the linearity assumption [19] but analysis of these and how they can be avoided goes beyond the scope of this paper.

There were few analyses that took place prior to clustering in order to assess the validity of the study.

1. We constructed three correlation matrices for each case and each subject (a total of 18 matrices) by calculating the correlation coefficients for each trial and averaging over the number of trials for each state. An initial difficulty that was raised when trying to conduct multi-subject analysis was the fact that the brains of the subjects were different. Despite this we followed the philosophy of [3] by averaging the activity in each ROI. In turn, we were interested if connectivity could be related to the different states and subjects. We performed the Network-based statistics with t-statistics threshold 3 and level of significance 0.05. Our design matrix for the Generalized Linear Model [6] was used in an effort to discriminate a) between the different cases and b) between different subjects. In a total of 5000 evaluations of the algorithm there were no significant results and therefore we concluded that there is no statistical difference between the matrices in terms of subjects and cases. Therefore we restricted our analysis by choosing data for each subject and for an average of trials in each subject instead of conducting a multi-subject and multi-trial analysis. In order to quantify differences, each correlation matrix was chosen using data from 3 time intervals: a) the first 4 for seconds where each subject was presented with a question b) the consequent 4 seconds where each subject was given a blank screen, and c) the last four seconds was exposed to an arrangement of symbols. Therefore we ended up with 6 matrices for each of these three segments.
2. We applied state-of-art algorithms in order to evaluate the modularity of our networks (defined later in the text). In specific, we executed the Louvain algorithm [20] for the correlation matrices that we considered for analysis and we found [mean 0.2963, standard deviation 0.0380]. These results raised questions as to whether our connectivity networks were hiding any form of organization. In fact this point discouraged us from investigating the network organization under co-affinity matrices where partitions with maximum modularity are being used in order to debunk hierarchical organization of networks. For additional affirmation of our results, we compared statistically our graphs with hierarchically nested random graphs by using the statistical z-score for average modularity [8]. Our graphs failed the test with a threshold of *z*_*t*_ = 2.3627 and confidence level of 1%. However, as will shall see later in the paper, this become the incentive to apply a “complementary” approach (using our algorithm) where instead of finding partitions with high modularity, we seek partitions with low modularity and we take the probability of two nodes being in the same cluster over all such partitions.

### Regularity partitioning algorithm

We continue by building the theoretical framework of the regularity partitioning algorithm. We begin by introducing the definition of a pseudo-random or *ϵ*–regular pair (or simply regular). We will use |*X*| to denote the cardinality of an arbitrary finite set |*X*|. We will use the notation *e*(*X*,*Y*) to denote the number of edges between sets *X* and *Y*. A bipartite graph with vertex clusters X and Y respectively we is said to be *ϵ*–regular [21, 22] if for every *A* ⊂ *X* and *B* ⊂ *Y*, with sizes |*A*| > *ϵ* |*X*| and |*B*| > *ϵ* |*Y*|, it holds that

|d(X,Y)−d(A,B)|<ϵ

where *d*(*X*,*Y*) (and *d*(*A*,*B*) respectively) stands for the fraction of the edges between the sets divided by the product of their cardinalities; of the corresponding sets, i.e., the quantity $d=\frac{e(X,Y)}{\left|X||Y\right|}$.

This definition is correlated with distribution of the number of edges between the pair.

Using Fact 1 (see appendix), by choosing a small *ϵ* and having in mind that density cannot be greater than 1, we can quantify the probability of edges connecting clusters of bipartite graphs. Therefore, if we can separate a graph into such randomly behaving pairs, we can have a probabilistic framework of the distribution of the edges and therefore of the existence of subgraphs in the graph. Formally, a partition of graph vertex set *V* of an arbitrary graph in *k* is clusters is called *ϵ*–regular (or simply regular) if it is equitable (every cluster has the same size) except perhaps a “junk” cluster *P*_0_ and:

- |*P*_0_| < *ϵ*|*V*|
- All but most *ϵ k* ^2^ of the pairs (*P*_*I*_,*P*_*j*_),1 ≤ *i* < *j* ≤ *k* are *ϵ*–regular.

The existence of such a partition for any graph has been proved in [9]. We mentioned at the introduction the impediments regarding the computationally efficiency of a potential algorithm that could decide if a partition of the vertex set of the graph can be *ϵ*–regular. Without diving into many technical details (that are beyond the scope of this paper), an efficient algorithm for obtaining such a partition can be found in the next lines. Note that the algorithm takes as input a) the graph under consideration, b) the *ϵ* parameter, c) the *l* parameter that defines the initial number of clusters, and d) the *h* parameter that restricts the clusters that are keep getting refined from having cardinality less than *h*.

The regularity partitioning algorithm for the graph *G*(*V*,*E*) and inputs *ϵ*, *l*, *h* is described as follows:

1. Obtain an initial partition by dividing the vertex set *V* into *l* + 1 clusters *P*_0_,*P*_1_,*P*_2_,…,*P*_*l*_. The cluster *P*_0_ is used in order to certify that the clusters *P*_*i*_, 1 ≤ *i* ≤ *l* will have equal sizes. Set current refinement number *k*_1_ = *l*.
2. If the size of the clusters becomes less than *h* then halt. Otherwise use the black-box algorithm to argue about whether pairs (*P*_*i*_,*P*_*j*_) are *ϵ*–regular If they are not, then return certificates.
3. If there are at most $\epsilon k_{i}^{2}$ *ϵ*–regular pairs, then halt and the current partition is an *ϵ*–regular partition and output that partition.
4. Otherwise, use the black box algorithm for refinement the partition further. This algorithm will result in a new partition with at most 1 + *l k*_*i*_ classes.
5. Set *k*_*i*+1_ = *l k*_*i*_ as the new refinement number, *Q* as the new partition under consideration, and *i* = *i* + 1 and repeat from step 2.

As the reader might observe, the algorithm keeps refining the obtained partition until the resulting partition is *ϵ*–regular.

The black-box algorithm for proving if a pair is *ϵ*–regular is defined in [14]. The outline of the algorithm is the following. For a bipartite graph, if there exists a set with significant deviation then three possibilities can take place: 1) the average degree of the graph will be small 2) there exists a set such that a small number of vertices have degree that deviates from the average degree in a specific way and 3) there are subsets in *A* and *B* such that their relative density exceeds a specific number.

The black-box algorithm for refining further a partition is also described in [14]. The outline of the algorithm is as follows: For the pairs that are not *ϵ*–regular return the certificates as above. Then partition the initial set *V* as follows: each certificate *A* should contain $\left\lfloor \frac{\left|A\right|}{m}\right\rfloor$ sets of vertices where *m* is $\left\lfloor \frac{\left|P_{i}\right|}{l}\right\rfloor$ and *l* is the initial refinement number *k*_1_.

When partitioning the collection of vertices in the initial partition and in the consequent partitions during the black-box refinement algorithm, we partition sequentially, *i*.*e*., having the vertices labeled as 1,…,|*V*| we start from 1, we select a set of size indicated by the current step of the algorithm, then we select the next set and so on. This way enables us to satisfy the disjoint property in the refinement algorithm and speeds up the computational time. In addition, a technical point worth mentioning is that each partition is associated with a “junk” cluster (usually indexed with 0) where the algorithm puts vertices that are not included in the partition. This can be viewed as a technical manipulation of the algorithm in order to keep the size of the clusters equal.

As we mentioned before, the aforementioned fact enables us to quantify the number of edges between *ϵ*–regular pairs. An example of this is a relevant result regarding the lower bound of the number of triangles. Following the spirit of [23], for any triple of subsets of the vertex set *V*, with (*X*,*Y*), (*Y*,*Z*), and (*Z*,*X*) are *ϵ*–regular (with densities *α*,*β*, and *γ* respectively), we calculate the number of triangles as:

(1−2ϵ)(α−ϵ)(β−ϵ)(γ−ϵ)|X||Y||Z|.

The previous formula tells us that in case of regular pairs, the number can be approximated from below the number of triangles in totally random tripartite graph. In the following experiment we count the number of triangles by randomly choosing 3 clusters (with uniform probability) from the regularity partition and calculating the lowest bound on the number of triangles out of 10 such repetitions.

Finally, the index of a partition *P* = *V*_0_ ∪ *V*_1_ ∪ … ∪ *V*_*k*_ can be defined as

$$ind(P)=\frac{1}{k^{2}}\sum_{V_{i},V_{j}\in P}(d{\left(V_{i},V_{j})\right)}^{2}.$$

The index of a partition can be viewed in a probabilistic way as the expectation of edges between the clusters of the partitions [24]. This quantity can be characterized as the energy of the partition in terms of how many edges exist in a pseudorandom graph and can be used for comparison between different partitions. An interesting fact is that when refining an *ϵ*–regular partition the index increases by a quantity that is a function of *ϵ* [21]. Therefore, the partition obtained in each step is a partition that has low modularity but increased index from the partition of the previous step. Interestingly enough, since the index is bounded above by 1, this idea leads to the proof of convergence of the algorithm. In addition, the algorithm allows us naturally to collect a family of partitions that have decreased modularity.

### Modularity and organization

An *ϵ*–regular partition can be seen as a collection of almost random bipartite graphs that approximate a network. It is therefore expected that the resulting partitioning of the algorithm will suffer from decreased modularity. In the work of Sales-Pardo et al. [8], the authors define a partition space of all partitions with maximum (local) modularity. In specific they consider the co-affinity between node *i* and *j* as the probability of these nodes ending up in the same cluster of a partition that has a local maximum modularity. We remind the reader that the modularity of a network partition *P* is defined as

$$MOD(P)=\sum_{i=1,..,m}\left[\frac{l_{i}}{L}-{\left(\frac{d_{i}}{2L}\right)}^{2}\right]$$

where *L* is the total number of edges, *l*_*i*_ is the total number of nodes within cluster *i*, *d*_*i*_ is the sum of degrees of all nodes inside cluster *i*, and the sum is over all *m* clusters of the partition.

Our approach complements the aforementioned approach as we define the probability of node *i* and *j* to belong to the same cluster over partitions with local minimum modularity. These partitions with decreased modularity stem from running our regularity partitioning algorithm. We construct the modified co-affinity matrix and we seek to develop a diagonal-block structure that will provide us with information of how the network is constructed in blocks of clusters that have high edge density between them and low edge density within. After constructing the modified co-affinity matrix, we use the reverse Cuthill-McKee algorithm [25] in order to move larger values to the diagonal and obtain a better perspective of the organization of the nodes.

One major application that stems from obtaining a regularity partition is the reduction of the number of nodes. In particular, suppose that we obtain a partition of the graph *G*(*V*,*E*) with k clusters and an exceptional set; *i*.*e*., *V* = *V*_0_ ∪ *V*_1_ ∪ … ∪ *V*_*k*_ where (*V*_*i*_,*V*_*j*_), *i*,*j* ∈ 1,…,*k* are regular pairs. Then the reduced graph of *G* is the graph with nodes that correspond to each cluster and edges take place only between regular pairs with density above *d*. It has been proved (we refer the reader to the Appendix for the corresponding theorem) that every graph properties of the original graph are mitigated to the reduced graph. Therefore, by reducing the data using regularity clustering allows us, up to a level of approximation, to contain all the information of the original data. In practice, when it is the case that *ϵ* is significantly small, we consider edges in the reduced graph between all the pairs and not only the pairs with density above *d* [14].

The final step after reducing the dimensionality of the network data is to apply well-known algorithms to the reduced data. An immediate advantage of this is from the computationally load efficiency perspective. In clustering techniques such as the Ncut algorithm [26] the main steps in obtaining the partition lie in computing the affinity matrix and, in turn, computing the eigenvalue decomposition. This can be extremely intensive for large datasets and therefore the advantage of our algorithm from that aspect is eminent. Using this approach, our philosophy was the following: We reduced the data after applying a regularity-partitioning algorithm by building the corresponding reduced graph. In turn, we applied the Ncut algorithm in order to obtain a clustering of the graph. We mapped this clustering to the original data and, eventually, we distributed the remaining points of the junk cluster to the closest clusters in order to obtain a final partition of all the vertices.

As we mentioned in the first section, we wish to compare this approach to the direct approach that applies the Ncut algorithm to the original data. As far as measuring the accuracy between the different approaches, a common measure used in the bibliography is the one that compares the ground truth labels of the data and the resulting labels obtained from the partitioning algorithm. Specifically, the accuracy is defined as

$$Accuracy=\left(\frac{\sum_{i=1}^{n}\sigma (y_{i},map(c_{i}))}{n}\right)$$

where *n* is the number of data points considered, *y*_*i*_ represents the truth label for point *i*, and *c*_*i*_ is the obtained cluster label for point *i*. The function *σ* equals is if its arguments are equal and 0 otherwise. The function *map* is a function that permutes the cluster labels to the points such that an optimal match is attained. This is usually executed via the Hungarian method [27].

A major problem in our case is the fact there is no ground truth for evaluating the accuracy of the clustering. Reducing the data and applying clustering techniques has been proved to work with better or similar accuracy than the application of the clustering techniques to the original data [14]. In our work we considered as ground truth labels the regions of interest identified in the experiment [2].

## Results

**Fig 1:**
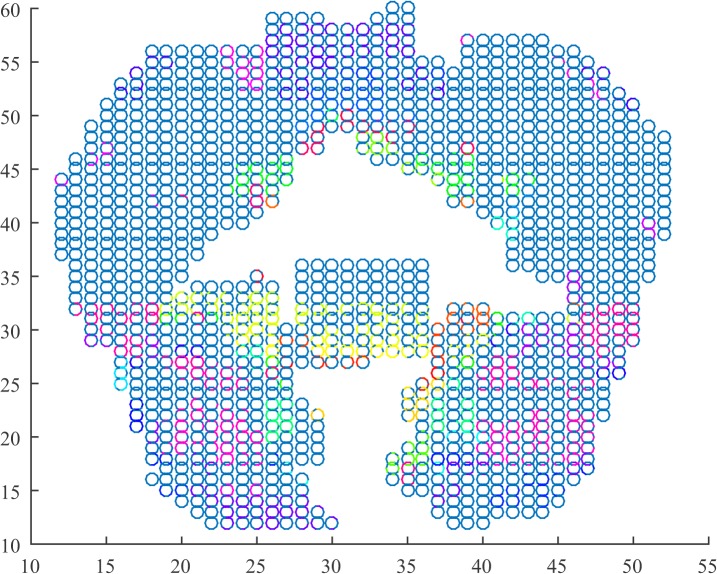
Cluster depiction in the coronal view (z = 0) of the cortex during question. Clusters identified by the regularity partitioning algorithm as seen on the coronal view obtained by the examined fMRI data. Each cluster is colored with a different color. During this epoch, the subject was exposed to a question. The stimulus was a sentence regarding an arrangement of symbols that would show up in the screen during the third epoch.

**Fig 2:**
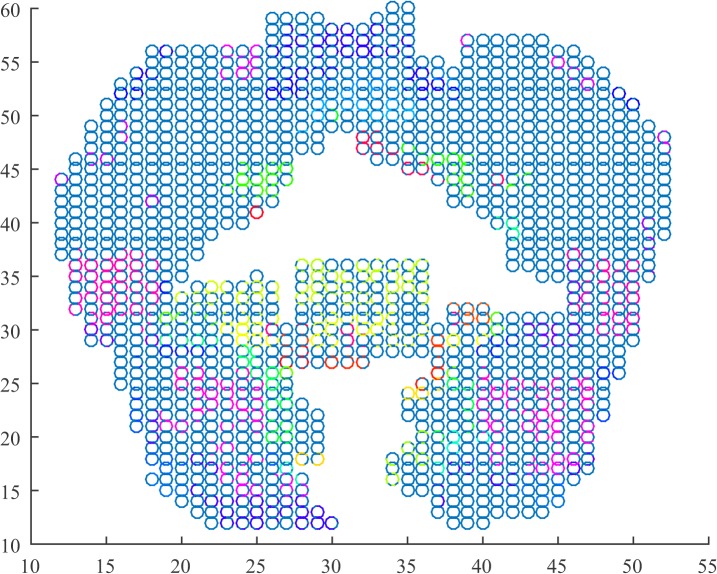
Cluster depiction in the coronal view (z = 0) of the cortex during blank screen. Clusters identified by the regularity partitioning algorithm as seen on the coronal view obtained by the examined fMRI data. Each cluster is colored with a different color. During this epoch, the subject was not exposed to a stimulus. Clusters seem to appear in the temporal and frontal lobes.

**Fig 3:**
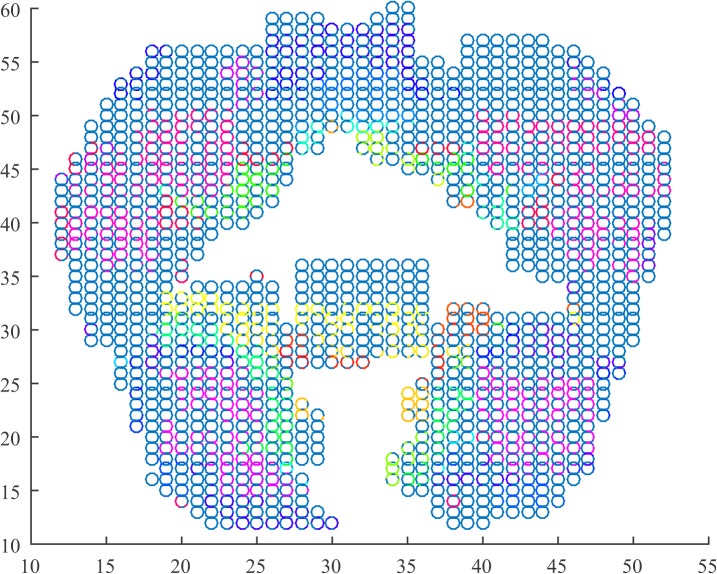
Cluster depiction in the coronal view (z = 0) of the cortex during symbol arrangement. Clusters identified by the regularity partitioning algorithm as seen on the coronal view obtained by the examined fMRI data. Each cluster is colored with a different color. During this epoch, the subject was exposed to the arrangement of symbols that was connected with the question in the first epoch.

We depict in Figs 1–3 for the first subject that the algorithm was able to identify. The parameters for the algorithm were set as *ϵ* = 0.016, *h* = 20, and *l* = 4. Each graph corresponds to a 4-second recording of fMRI. We used the locations of the data associated with the voxels as well as a scatter plot to depict the coronal view of the brain. In turn, we used a MATLAB colormap to assign each cluster to each a different color.

**Fig 4:**
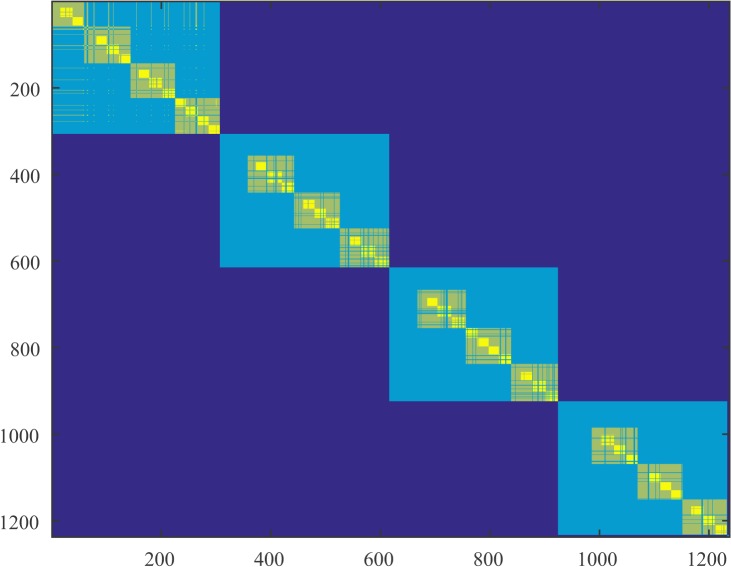
Modified co-affinity matrix during question. Modified co-affinity matrix for subject 1 during the segment that the subject was exposed to a question. We display only the first 1200 vertices. The lighter the color, the higher the probability two nodes belong to the same cluster over all the regularity partitions obtained by our algorithm, *i*.*e*., partitions with decreased modularity. Nodes were labeled properly such that the weight in the diagonal is higher using an optimization algorithm.

**Fig 5:**
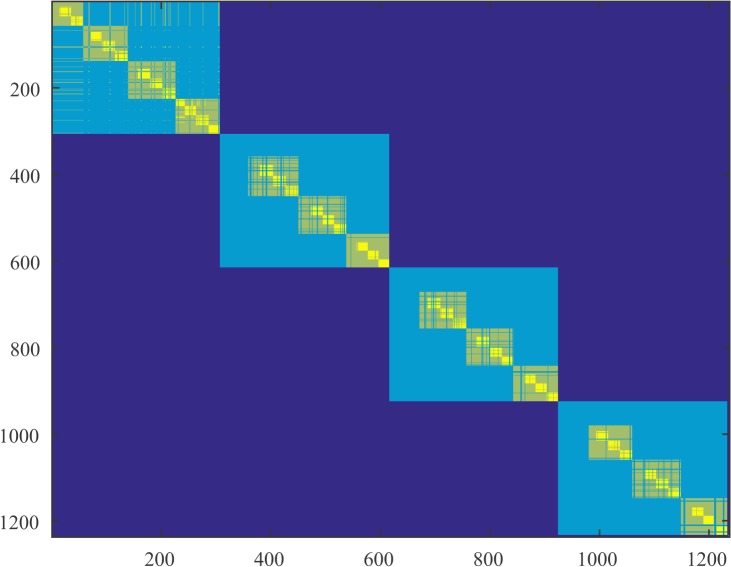
Modified co-affinity matrix during question blank state. Modified co-affinity matrices for subject 1 during the segment that the subject was exposed to a blank screen after the question. We display only the first 1200 vertices. The block diagonal organization seems to be segmented; vertices are now distributed in different clusters.

**Fig 6:**
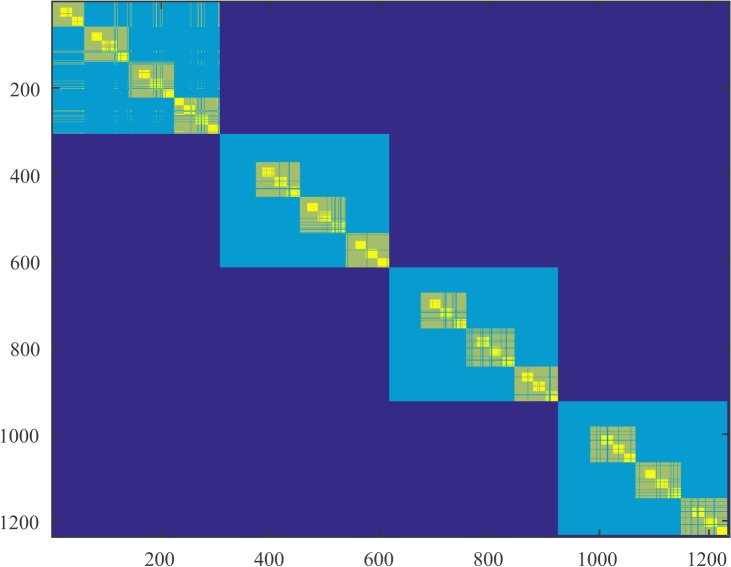
Modified co-affinity matrix during symbol arrangement. Modified co-affinity matrix while the subject was exposed to the arrangement of symbols that was connected with the question in the first epoch. The segmentation of the block diagonal organization seems to be segmented in this time frame too.

In Figs 4–6 we present parts of the modified co-affinity measure that we introduced for subject 1 and for the 3 segments.

Throughout the three different epochs, we observe differences on the formation of the clusters mainly on the temporal and frontal lobes. It seems that during the first epoch, the clusters in both subjects seem to lie topographically in similar positions with few clusters lying on the coronal view of the frontal and the temporal lobe. As the trial progresses, we observe emerging clusters in these two areas of the brain as well as in the intra-hemispherical connection between the two temporal lobes. From a functional point view, we examine the number of clusters, the number of triangles as well as the index of the resulting partition in each epoch and subject. The summary of the results is tabulated in Table 1.

**Table 1:** Regularity partitions’ characteristics.

Segments	Number of Clusters	Size of Cluster	Lo. bound on triangles	Index
1^st^	(133, 6)	(18, 0)	(6, 11)	(0.0426, 0.0404)
2^nd^	(137, 13)	(18, 0)	(12, 17)	(0.0434, 0.0432)
3^rd^	(138, 9)	(18, 0)	(9, 14)	(0.0427, 0.0401)

Characteristics of the obtained regularity partitions for each segment and averaged over the six segments of the experiment. Data is presented in the form of (mean, standard deviation) and rounded to the smallest integer (except index). The segments correspond to the time frame that the subject was exposed to question, the time frame that there was a blank screen, and the time frame that the subject was exposed to an arrangements of symbols regarding that question. We present the number of clusters alongside their sizes. In addition, we calculated the lower bound for the number of triangles and the expectation of the edges in each partition. We refer the reader to the main body of the article for explanation of these quantities.

We observe that the number of clusters increases during the period after the subject is exposed to the object (segments 2 and 3). It also evident from the co-affinity matrix that during segments 2 and 3 there exists an increased segmentation of the diagonal blocks meaning that the probability of two nodes ending up in the same cluster is decreased for a large population of nodes. In terms of regularity this means that nodes are most likely to end up in different clusters where the distribution of edges is higher. This can account for a raw interpretation of the effective organization of the brain that we mentioned in the abstract. Nodes tend to increase modularity in terms of not intra-cluster but inter-cluster connectivity. This can be confirmed by the slight increase in the index of the partition. We also observe a small increase in the lower bound on the triangles. It is interesting to note here that triangles can be measured between any cluster of the regularity partitioning since all the clusters are pairwise regular. Therefore any increase in the number of triangles regards the whole cortex and not only, for example, clusters that are topologically adjacent.

From a computational load point of view, our algorithm runs extremely fast as it reduced thousands of voxels to a few hundred data points. As far as accuracy is regarded, Table 2 reveals that for all the segments our algorithm identifies the ground truth labels with accuracy very close to the Ncut algorithm (2.6% deviation of the mean efficiencies for the two approaches averaged over the three segments). This can enhance processing thousands of voxels in high-dimensions fMRI images without losing significant information about voxels’ interdependences.

**Table 2:** Comparison of the regularity partitioning-based cluster method to the Ncut method.

Segments	Reduced-Ncut	Normal-Ncut
1^st^	(0.9016, 0.0098)	(0.9245, 0.0031)
2^nd^	(0.8987, 0.0108)	(0.9241, 0,0026)
3^rd^	(0.9003, 0.0132)	(0.9238, 0.0032)

Comparison of the accuracy for the two methods of clustering: For the Reduced-Ncut, we initially applied the regularity partitioning algorithm to obtain a reduced representation of the data. In turn, we applied the Ncut algorithm to the reduced data and we mapped the result back to the original data. For the Normal-Ncut method, the Ncut the algorithm was applied directly to the data. All data is presented as (mean, standard deviation) for the three segments and averaged for the six subjects of the experiment. We refer the reader to the text for additional details that pertain to construction of the reduced graph.

## Discussion

As we mentioned in the Materials and Methods section, an *ϵ*–regular partition is a partition that consists of *ϵ*–regular pairs. From a probabilistic point of view, a vertex in a regular pair has a probability proportional to the density of the pair and this hold for every subset of reasonable size. From a functional point of view, regular pairs can play the role of robust functional centers in the brain where information can travel between them and between subsets arbitrary subsets with high probability if *ϵ* is chosen small such as in this study. During the time frames after the subjects were exposed to the stimulus, the clusters of the inspected part of the brain that were identified from the algorithm were densely connected. This argument is partially corroborated by the fact that the lower bound of the number triangles is significant as well as from the fact that the index of the partition is increased. In addition, clusters seem to appear in the temporal and frontal lobes. Co-affinity matrices also revealed a block diagonal organization that seemed to break when the subjects were thinking during segments 2 and 3. This re-organization increases the expected number of edges between the clusters and therefore enhances the functional connectivity between distant and not distant pairs. Finally, clustering the reduced data seems to provide accurate results compared to direct clustering; a fact that ameliorates the computational load of many standard clustering techniques when it comes to massive brain data.

## Conclusions

In this paper we applied a famous theoretical graph partitioning result using a modified algorithm. The basis of this result lies on identifying pseudorandom pairs in dense graphs. These pairs, by manipulating the *ϵ* properly, can behave as close to random as possible. In addition this can take place for any sufficient large subset of nodes within their body. Under these remarks, we calculated the *ϵ*–regular partitions for the fMRI data of six subjects that were exposed to stimuli. We observed that after exposure to the stimuli the brain exhibited an enhanced organized behavior; the number of triangles constructed was increased compared to the blank epoch where no stimulus was presents. This may construct the basis of different discriminant for identifying cognitive states. Additional data should be used to strengthen the result. We strongly believe though that pseudorandomness can quantitatively depict these deviations in the different functional brain networks in terms of how networks are organized to transmit information efficiently.

## Appendix

### Regularity

We prove the following fact regarding the probability of a vertex having a neighbor in a regular pair. We will use the notation *e*(*a*,*Y*) to denote the number of edges between as single vertex *a* and a set *Y*.

#### Fact 1

Consider an *ϵ*–regular pair (*A*,*B*). For any subset *Y* ⊂ *B*, with |*Y*| > *ϵ*|*B*|, the size of the subset of *A*, *X* which has the property that for any *v* ∈ *X*, *e*(*v*,*X*) > |*d*(*A*,*B*) − *ϵ*| |*Y*| has size strictly greater than *ϵ*|*X*|.

#### Proof

Consider the probabilistic event of an edge between vertices equipped with the uniform measure. Then the probability of observing edges between the vertex *v* and the subset |*Y*| with the specification as described in the remark, is $\frac{e(v,Y)}{\left|Y\right|}$. Summing for all *v* ∈ *X* and diving by the cardinality |*X*| results in the density *d*(*X*,*Y*). By the premises, this has to be strictly greater than |*d*(*A*,*B*) − *ϵ*|. But since (*A*, *B*) is an *ϵ*–regular pair, this holds for any subset of size |*X*| > *ϵ* |*A*|.

### Reduced graph

Regarding reducing the data, consider a graph *R*(*V*,*E*) and an integer *t*. Let *R*(*t*) be the graph obtained from *R* by replacing each vertex in *V* by a set *V*_*x*_ of *x* independent vertices, and joining *u* ∈ *V*_*x*_ and *v* ∈ *V*_*y*_ if and only if (*x*,*y*) ∈ *E*.

We used the following theorem that shows us how to use the reduced graph of an arbitrary graph.

#### Theorem

Given *d* > *ϵ* > 0, a graph *R* and a positive integer *m*, construct a graph *G* following these steps:

- Replace every vertex of *R* by *m* vertices.
- Replace the edges of *R* with regular pairs of density at least *d*.

Let *H* be a subgraph of *R*(*t*) with *h* vertices and maximum degree Δ > 0, and let *δ* = *d* − *ϵ* and *ϵ*_0_ = *δ* ^Δ^/(2 + Δ). If *ϵ* ≤ *ϵ*_0_ and *t* − 1 ≤ *ϵ*_0_ *m*, then *H* is embeddable into *G* (*i*.*e*., *G* contains a subgraph isomorphic to *H*). In fact,

$$\|H\to G\|>{\left(\epsilon_{0}m\right)}^{h}$$

where ‖*H* → *G*‖ denotes the number of labeled copies of *H* in *G*.

Practically this lemma involves three graphs, *R*(*t*), *G*, and *H*. Certain density constraints are imposed to *R*(*t*) in order to contain a subgraph *H*. Since *R*(*t*) is a subgraph of *G*,*H* will be contained in *G* too and therefore constructing a reduced graph allows us to mitigate patterns from the reduced to the original graph. In specific, if *t* and *d* are appropriate with respect to *ϵ* and Δ, it is possible to find a graph *H* with small number of vertices but with high number of edges. Copies of these graphs can be injected into *G* by careful construction.

### Index of a partition

We will use *X* × *Y* to denote the Cartesian product between two arbitrary sets *X* and Y. The characteristic function *χ*(*A*) of a set takes the value 1 when the element under consideration belongs to *A* and 0 otherwise.

As we mentioned in Fact 1, the density *d*(*X*,*Y*) between two clusters *X*,*Y* can be viewed as the union of the probabilistic events of the edges between the two clusters:

$$\rho (e(X,Y))=\rho (\cup_{x,y\in e(X,Y)}\rho (x,y))=\sum_{e(X,Y)}\rho (X,Y)=\frac{1}{\left|X||Y\right|}e(X,Y).$$

Similarly, if we partition the sets X and Y into *X*_1_, *X*_2_ and *Y*_1_, *Y*_2_ then we can write the Cartesian product as (*X*_1_ × *Y*_1_) ∪ (*X*_1_ × *Y*_2_) ∪ (*X*_2_ × *Y*_1_) ∪ (*X*_2_ × *Y*_2_) and therefore we can define densities on each part. For example for *X*_1_ × *Y*_1_, we can define

$$\rho_{X_{1}\times Y_{1}}(e(X,Y)\cap (X_{1}\times Y_{1}))=d(X_{1},Y_{1}).$$

This can be extended to a partition of a arbitrary size trivially. Consequently, we can see that each density corresponds to a pair of clusters can be viewed as a probability measure of the edges between the pair. Now we can define the function *f*: *X* × *Y* → *R* as *e*(*X*,*Y*). Analogously, we define this function restricted on refinement *P* with k clusters

$$f=\sum_{\chi (X_{i}\times Y_{j})}e(X_{i},Y_{j})$$

where *χ* is the characteristic function of the set and refers to the *X*_*i*_ and *Y*_*j*_. Now a crucial point is to consider the 2-norm of this function in the space of the discrete square integrable functions. The square norm of this function restricted in the partition *P* is

$${\left\|f_{P}\right\|}_{2}^{2}=\sum_{\chi (X_{i}\times Y_{j})}{\left(e(X_{i},Y_{j})\right)}^{2}\;\rho_{X_{i}\times Y_{j}}$$

where *ρ* is defined as before. This quantity is exactly the expectation of the edges in this space of functions and corresponds to the index of the partition.
